# Physicochemical and Antimicrobial Characterization of Chitosan and Native Glutinous Rice Starch-Based Composite Edible Films: Influence of Different Essential Oils Incorporation

**DOI:** 10.3390/membranes13020161

**Published:** 2023-01-27

**Authors:** Karthikeyan Venkatachalam, Natthida Rakkapao, Somwang Lekjing

**Affiliations:** 1 Faculty of Innovative Agriculture and Fishery Establishment Project, Prince of Songkla University, Surat Thani Campus, Makham Tia, Mueang, Surat Thani 84000, Thailand; 2 Department of Applied Chemistry, Faculty of Science and Industrial Technology, Prince of Songkla University, Surat Thani Campus, Makham Tia, Mueang, Surat Thani 84000, Thailand; 3 Center of Excellence in Membrane Science and Technology, Faculty of Science, Prince of Songkla University, Hat Yai Campus, Hat Yai, Songkhla 90110, Thailand

**Keywords:** chitosan, native glutinous rice starch, essential oil, edible film, physicochemical properties, antimicrobial activities

## Abstract

Biopolymer-based edible packaging is an effective way of preserving food while protecting the environment. This study developed an edible composite film using chitosan and native glutinous rice starch (NGRS) and incorporated essential oils (EOs) such as garlic, galangal, turmeric, and kaffir lime at fixed concentrations (0.312 mg/mL) to test its physicochemical and antimicrobial properties. The EO-added films were found to significantly improve the overall color characteristics (lightness, redness, and yellowness) as compared to the control film. The control films had higher opacity, while the EO-added films had slightly reduced levels of opacity and produced clearer films. The tensile strength and elongation at break values of the films varied among the samples. The control samples had the highest tensile strength, followed by the turmeric EO-added samples. However, the highest elongation at break value was found in the galangal and garlic EO-added films. The Young’s modulus results showed that garlic EO and kaffir lime EO had the lowest stiffness values. The total moisture content and water vapor permeability were very low in the garlic EO-added films. Despite the differences in EOs, the Fourier-transform infrared spectroscopy (FTIR) patterns of the tested films were similar among each other. Microstructural observation of the surface and cross-section of the tested edible film exhibited smooth and fissureless patterns, especially in the EO-added films, particularly in the galangal and kaffir lime EO-added films. The antimicrobial activity of the EO-added films was highly efficient against various gram-positive and gram-negative pathogens. Among the EO-added films, the garlic and galangal EO-added films exhibited superior inhibitory activity against *Escherichia coli*, *Salmonella* Typhimurium, *Listeria monocytogenes*, *Staphylococcus aureus*, and *Pseudomonas fluorescence*, and turmeric and kaffir lime EO-added films showed potential antimicrobial activity against *Lactobacillus plantarum* and *L. monocytogenes*. Overall, this study concludes that the addition of EOs significantly improved the physicochemical and antimicrobial properties of the CH-NGRS-based edible films, making them highly suitable for food applications.

## 1. Introduction

Recent studies have focused on developing eco-friendly, edible, and biodegradable films [1]. In addition, the field of bio-based polymers made from various agricultural commodities and/or food waste products has shown a growing interest in developing materials capable of film-forming and antimicrobial properties that contribute to food safety and shelf life [2,3]. Normally, for developing biobased edible films, materials, particularly polysaccharides, lipids, and proteins, are used as they are from natural resources and are considered highly safe [4]. The main properties of edible films include colorants, antimicrobials, and bioactive contents. Besides their edibility and biocompatibility, edible films’ advantages are their aesthetic appearance, barrier properties to gases, non-toxic, non-polluting nature, and low production cost. Edible films are used to preserve food products from several extraneous factors. They are thin layers of material layered or covered on the food product [5,6]. Polysaccharides are among the most widely used edible biopolymers as they are primarily obtained from nature and have low to no hazardous effects on food products. Starch-based films have been widely employed in food and non-food applications [7]. Most native starch is found in carbohydrates, which play a key role in human diets and exist in plants as granular structures of varying sizes and shapes. There are two glucan molecules in native starches, amylose and amylopectin. Compared to normal starch, native starch contains a high proportion of amylose and a low proportion of quickly digestible starches. The primary purpose of starch-based films is to prevent food from losing taste, color, flavor, or appearance [8]. Despite their potential, starch-based films lack mechanical properties, such as brittleness and low tensile strength, which prevent them from replacing other biopolymers, such as proteins and lipids [9]. In recent decades, composite films have been used in various applications, including food packaging. This is due to the fact that composite films have the potential to bridge two distinct types of biopolymers, each with its characteristics but, when combined, these biopolymer-derived films can protect a variety of food products as a whole [10]. An influential polysaccharide produced from crustacean shells is chitosan, a well-known polysaccharide considered non-toxic, biodegradable, and a multifunctional polysaccharide that forms films and exhibits effective antimicrobial activities against gram-positive and gram-negative bacteria, as well as mold and growth [11]. Many biopolymers and additives have been combined with chitosan to enhance the film’s properties and provide food with strong barrier properties against various drawbacks, particularly microbial growth.

Packaging with antimicrobial properties is one of the most promising active packaging systems for killing or inhibiting spoilage and pathogens contaminating food. Despite the fact that chitosan possesses excellent antimicrobial properties due to its polycationic characteristics, its beneficial antimicrobial properties may vary according to its deacetylation levels, pH values, molecular weight, matrix, and microorganisms [12]. Adding or encapsulating microbial-resistant substances, such as essential oils, in edible films is therefore necessary, and this has been recommended by many researchers to improve the quality of food products and retard the growth of microorganisms for an extended period [13]. Generally, the antimicrobial activity of essential oils (EOs) depends on the presence of phenols and terpenes. As a result of the synergistic effect between EOs and their constituents, edible films that incorporate EOs are made with enhanced functional properties [14]. The shelf life of food is increased, especially for foods with high fat levels. Moreover, the United States Food and Drug Administration (FDA) has classified EOs as generally recognized as safe compounds (GRAS) and has approved their use as food additives or flavorants [15]. It is important to note that EOs derived from different sources may have different properties and display different mechanisms of action on pathogens. Garlic essential oil (GO) has been commonly used in medicine since ancient times. It has been widely reported to exhibit several health-beneficial properties, including antimicrobial, antiviral, and fungi-toxic properties. It is well established that the beneficial effects of GO are primarily attributed to sulfur compounds, such as allicin, diallyl disulfide, and diallyl trisulfide [16,17]. Galangal (*Alpinia galanga* Linn.) is primarily used as a flavoring and medicinal spice. The essential oil of galangal consists of 1, 8-cineole, and other oxygen-containing monoterpenes. It has been extensively studied for its antioxidation, anti-tumor, anticoagulation, anti-inflammatory, and antibacterial properties. The application of galangal essential oil on food products effectively combats food poisoning bacteria, particularly *S. aureus* [18,19]. Throughout Asia, turmeric is used extensively to enhance color and flavor in various foods. In traditional and modern medicine, turmeric has been found to be anti-inflammatory, anti-HIV, antibacterial, antioxidant, anti-diabetic, and anti-cancerous [20]. Major components of turmeric oil are curcumin, polyphenols, terpenes, terpenoids, and sterols, which provide similar benefits to turmeric. Studies have shown that turmeric oil contains phenolic content that inhibits microbial growth in starch-based edible films [21]. The kaffir lime leaf is another important herb widely used for flavoring, and its essential oil contains several active ingredients, including citronellal, citronellol, linalool, and geraniol. The essential oil of the kaffir lime leaf has the ability to inhibit pathogenic microorganisms for food poisoning, including *Salmonella* and Enterobacteriaceae [22,23]. In spite of the fact that there are numerous studies available on edible films that integrate essential oils, most are carried out on an individual basis and/or compare different concentrations of that particular essential oil. This study aimed to develop a composite edible film using chitosan (CH) and native glutinous rice starch (NGRS), and incorporate it with different sources of essential oil at a fixed concentration as an active ingredient. The resulting films were then evaluated for their various physicochemical, microstructural, and antimicrobial properties.

## 2. Materials and Methods

### 2.1. Materials and Film Formation

The food grade chitosan powder (200 mesh particle size, 80% degree of deacetylation, MW = 8.97 × 10^5^ Dalton) was purchased from Sinudom Agriculture Products Co., Ltd., Surat Thani, Thailand. The native glutinous rice starch (NGRS) was purchased from Thai flour Industry Co., Ltd., Bangkok, Thailand. Glycerol, Tween 80, and 85% lactic acid were purchased from J. T. Baker (NJ, USA). Pathogenic bacterial cultures such as *Escherichia coli* (DMST 15537), *Salmonella* Typhimurium (DMST 15674), *Listeria monocytogenes* (DMST 23145), and *Staphylococcus aureus* (DMST 8840) were purchased from the Department of Medical Sciences, Ministry of Public Health, Nonthaburi, Thailand. *Pseudomonas fluorescens* (TISTR 358) and *Lactobacillus plantarum* (TISTR 543) were purchased from the Thailand Institute of Scientific and Technological Research, Pathum Thani, Thailand. The edible essential oils used in this study were purchased from Thai China Flavors and Fragrances Industry Co., Ltd., Bangkok, Thailand. All the media for microbial analysis were purchased from HiMedia (Mumbai, India).

Chitosan film was formed in accordance with the method of de Souza Soares et al. [24] with modifications. To form the control film, 1.2075 g of chitosan was mixed well in a beaker containing 100 mL of lactic acid (0.12 M/L) and, similarly, 1.2148 of NGRS was mixed well in a beaker containing 30 mL of lactic acid (0.12 m/L). After that, in another beaker (250 mL), the chitosan and NGRS solutions were combined, followed by the addition of 0.2% tween 80 (*v*/*v*) and 0.5% glycerol (*v*/*v*), and then the film-forming mixture was thoroughly mixed well. After that, the film formation mixture was placed on a magnetic stirrer with a hot plate, and the mixture was heated between 85 and 90 °C, and continuously stirred for 30 min. After the heat treatment, the film-forming solution temperature was brought down to 27 ± 2 °C. Then, the film was cast in a Petri dish (10 cm in diameter) and then dried in a hot air oven at a fixed temperature (50 °C) for 5 h, and then dried films were carefully peeled off from the Petri dish. For the essential-oil-added film preparation, all the above procedure was the same, except the addition of 0.312 mg/ mL of essential oil (EO) was introduced in the film-forming solution after the addition of glycerol and all the remaining procedure continued further as mentioned above. Then, all the dried films were stored in a low-density polyethylene Ziplock bag and placed in the desiccator at 27 ± 2 °C at 50% relative humidity. The films were utilized for the following analysis within a week after being manufactured.

#### 2.1.1. Color Characteristics, Opacity, and Appearance

The color characteristics (lightness (L*), redness (a*), and yellowness (b*)) values of the tested films were evaluated using a Hunter Lab colorimeter [MiniScan EZ, HunterLab, Reston, VA, USA].

Film opacity [25] was determined using the following equation based on the film absorbance at 600 nm and the film thickness:

(1) $$Opacity=\frac{A}{X}$$

In this equation, A represents the film absorbance at 600 nm, and x represents the film thickness (mm). The film opacity was expressed as A600/mm.

For the film appearance, using a handheld digital camera [Coolpix B500, Nikon, Tokyo, Japan], a digital image of the tested edible films was taken.

#### 2.1.2. Thickness

A handheld digital micrometer (Mitutoyo 293-340-30 External Micrometer, Kawasaki, Japan) was used to measure the thickness of the tested films following the method of Keawpeng et al. [26]. A total of ten films were used per treatment, and each film was tested for thickness at six random areas. Results are expressed in millimeters.

#### 2.1.3. Textural Properties

A texture analyzer was used to measure the films’ tensile strength, elongation at break (EAB), and Young’s modulus [27]. Before analysis, the films were cut into 2 × 6 cm samples, and the tensile strength was determined at a speed of 0.30 mm/s with a 0.1 N preload. Measurement ended when the film was separated into two pieces. The tensile strength of a film is determined by the ratio of the maximum load to its cross-sectional area. The results are expressed in MPa. Film EAB was calculated by dividing the film’s initial length (6 cm) by its break length, and the results are expressed as a percentage. Young’s modulus was calculated based on the stress–stress curve, and the results were expressed as MPa.

#### 2.1.4. Moisture Content and Water Vapor Permeability

For moisture content, the initial weight (Wo) of tested film (50 × 20 mm) was obtained by using a digital weighing balance (Sartorius, BSA2245-CW, Göttingen, Germany) and then drying it at 105 °C to obtain a constant weight (W1). After that, the following equation was used to calculate the film’s moisture content, and the results were expressed in percentages.

(2) $$Moisture\;content\;\left(\%\right)=\frac{W_{0}\times W_{1}}{W_{0}}\;\times \;100$$

Water vapor permeability was evaluated using the gravimetric modified cup method of Pereda et al. [28]. During the tests, cylindrical test cups (40 mm in diameter) were filled with 35 mL of distilled water to ensure 100% relative humidity. Afterward, the film was applied to the cup, the cap provided a taut surface and sealed the sides, and the set-up was left to allow the water vapor to permeate. The initial weight was recorded, and then the cups were placed in a pre-equilibrated desiccator (10–25% relative humidity) using silica gel. Every 2 h for 24 h, the cup weight, relative humidity, and temperature were recorded. The following formula was used to calculate the water vapor permeability (WVP) of the film:

(3) $$WVP\;\left(Kg\;Pa^{-1}s^{-1}m^{-1}\right)=\left(G\;\times \;X\right)[\;t\;\times \;A\;\times \;S\;\times \;\left(R_{1}-R_{2})\right]$$

where G represents the water vapor flow (kg), x the film thickness (m), t the time (s), A the area (m^2^), S the saturated water vapor pressure (Pa) at the measured temperature, R1 the relative humidity in the cup, and R2 the relative humidity in the desiccator.

#### 2.1.5. Microstructural Analysis

The microstructure of the films was examined using a scanning electron microscope (SEM, SU3900, Hitachi, Ltd., Tokyo, Japan) following a method similar to the one described by Zhang et al. [25] with some modifications. In this regard, the films were prepared in two parts, including surface section and cross-section; both samples were cryofractured in liquid nitrogen and, then, a thin layer of gold was coated on the film samples for 5 min using a sputter coater. After that, the film samples were observed under an SEM (15 kV for the surface section and 5 kV for the cross-section) with a magnification of 1000.

#### 2.1.6. FTIR

A method of Bhatia et al. [29] with some modification was used to examine the FTIR spectroscopy (Thermo Electron Corp., Madison, WI, USA) of the tested edible films. The films were mixed in a 1:100 ratio with potassium bromide, and the mixture was ground into powder and pressed into pellets. The films were then scanned to determine possible interactions between essential oil, glycerol, chitosan, and NGRS, and the influences of processing conditions, by scanning 32 times within the wavelength range of 500–4000 cm^−1^ with 2 cm^−1^ resolution for each recorded spectrum.

#### 2.1.7. Antimicrobial Analysis

The antimicrobial analysis of the tested films was done by following the method of Amankwaah et al. [30]. The dried films were used for testing the antimicrobial activity against *E. coli*, *S.* Typhimurium, *L. monocytogenes*, *S. aureus*, *P. fluorescens*, or *L. plantarum*. The pathogenic bacterial strains were individually cultured by transferring a loopful of bacterial strains using an inoculation loop into the 20 mL sterile broth (tryptic soy broth (TSB)). After the inoculation, the broth was kept in the incubator for 24 h at 37 °C. Then, a loopful of each revived bacteria from the broth was again transferred separately into tryptic soy agar (TSA) slants and incubated again for 24 h at 37 °C. After the incubation, the slants with bacteria were refrigerated (4 °C) and used as stock cultures. Before each experiment, the loopful of bacterial strains was taken from the respective bacterial slants and cultured in sterile TSB for 24 h at 37 °C. A suspension of bacteria containing approximately 10^7^ CFU/mL was prepared in TSB from overnight cultures and used for antibacterial testing. In sterile plates, film samples (2 cm by 2 cm) were cut into small pieces, and bacterial suspensions (10^7^ CFU/mL) were added. A control culture was one without a film and another with a chitosan–NGRS film but without essential oil. At the optimal temperature of each microorganism, the plates were incubated and shaken for 24 h at 60 rpm in an incubator shaker. From this, samples were collected at intervals of 0, 2, 4, 8, 12, 24, and 48 h, and diluted with 0.1% of 9 mL peptone solution. After the appropriate dilutions were made, 1 mL of dilution from each microbial culture was separately poured and spread into the plates, which had the specific agar relevant to the microorganism; for the *L. monocytogenes*, *S. aureus,* and *P. fluorescens*, brain heart infusion agar-based plates were used, and for the *E. coli*, *S.* Typhimurium, and *L. plantarum*, tryptic soy agar-based plates were used. After inoculation, the plates were incubated for 48 h at 37 °C. The antimicrobial activities of the films were identified by checking on the microbial count, and the results were expressed as a long colony-forming unit (CFU/mL).

#### 2.1.8. Statistical Analysis

All the analyses were done in triplicate, and the results are presented in mean values and standard deviations. One-way analysis of variance (ANOVA) and the Duncan multiple range test with a significant value of 0.05 were applied to identify the significant differences among the mean values. This study used the SPSS statistical software by International Business Machine (IBM, Armonk, NY, USA) (version 6 for windows) to run the statistical analyses.

## 3. Results

### 3.1. Color Profile, Opacity, and Appearance

**Figure 1:**
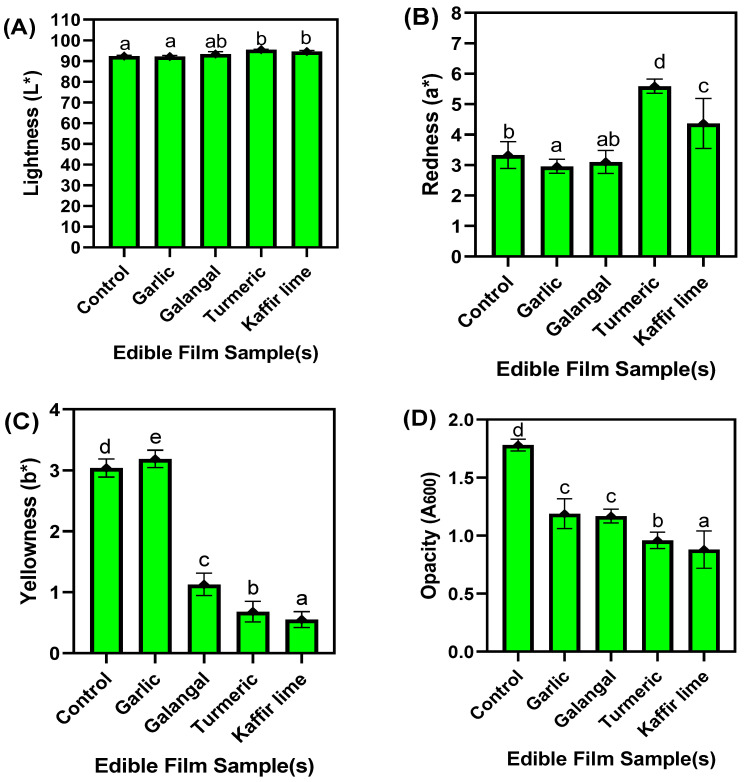
Color profile (**A**–**C**) and opacity (**D**) of chitosan–NGRS based edible films incorporated with and without EOs. Note: The different letters shown in the figure represents significant differences between means.

**Figure 2:**
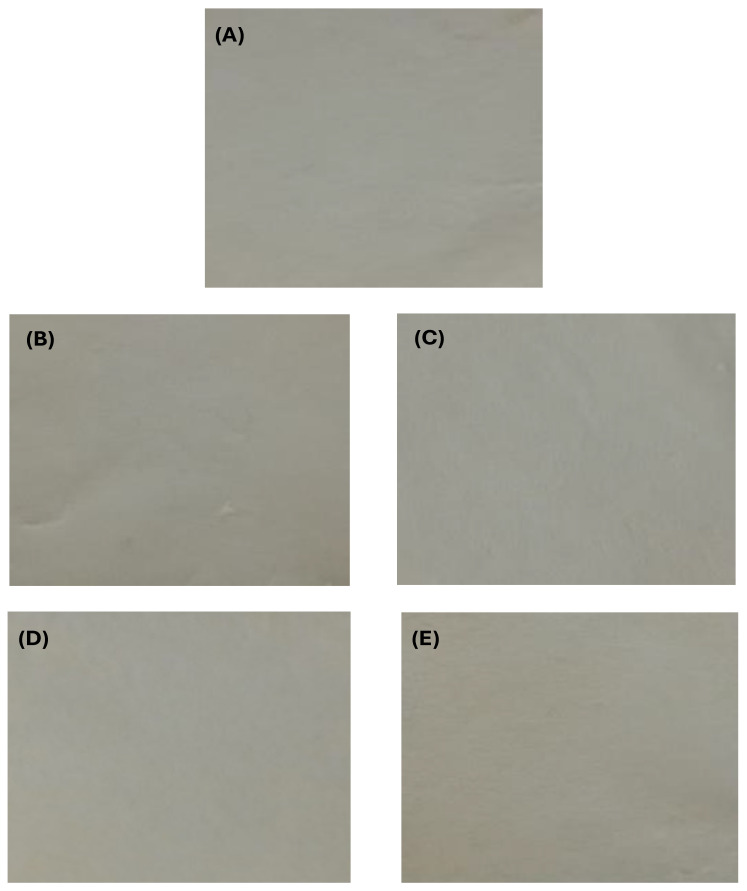
Appearance of chitosan–NGRS based edible film incorporated with and without EOs. Note: The different letters shown in the figure represents significant differences between means. (**A**) represents control film; (**B**) represents garlic oil added film; (**C**) represents galangal oil added film; (**D**) represents turmeric oil added film; (**E**) represents kaffir lime oil added film.

The color profiles, including lightness, redness and yellowness, opacity, and the appearance of the edible films made of chitosan and NGRS and incorporated with various EO, were tested, and the results are given in Figure 1 and Figure 2. Among the film samples, the film’s color profile differed somewhat (Figure 1A–C). The lightness of the tested edible films exhibited similar values and represented an insignificant influence on the light transmittance and color properties, despite the EOs used. Among the samples, turmeric and kaffir lime EO-based films had transmitted slightly lighter as compared with the other samples; however, the overall light transmittance values of the films were not adversely affected. On the other hand, the redness and yellowness values of the films were significantly affected by the EOs. The results showed a significant correlation between redness and yellowness; particularly, an increase in redness level in the film adversely reduced the yellowness values and vice versa. This study found that a higher level of redness in the films was noticed when incorporating turmeric EO and kaffir lime EO. In contrast, the yellowness values were predominant in the films containing garlic and galangal EO. The control films had the highest yellowness compared to other samples with EOs. Olawuyi and Lee [31] reported that variations of color values in the biopolymer edible films resulted from the natural color of the plant-based additives added to the composition. Socaciu et al. [32] found that the application of heat during film formation significantly affected the overall color qualities, and their study found that the application of high temperature (>85 °C) in the film formation had darker and more yellowish color effects. This is in accordance with the present study. On the other hand, the opacity of the films significantly varied among each other (Figure 1D). Generally, the films’ opacity represents the light emission restriction. A higher opacity level indicates a lower degree of transparency [33]. Basiak et al. [34] suggested that adding starch-based polymers in the edible film composition could adversely affect the film’s opacity, due to the formation of dense aggregation occurring in the amylose-rich starches. This study found that a higher level of opacity was noticed in the chitosan/starch-based film without EO, followed by the films with EOs. Among the different EO-added films, the garlic and galangal samples did not show significant differences. Additionally, the kaffir lime EO-added films were slightly better than the others, having the least opacity, followed by the turmeric EO-added films. Overall, the EO-added films improved transparency by lowering film opacity. Atarés and Chiralt [35] reported that adding EOs could induce the miscibility of biopolymer blends and thus decrease the film opacity. 

Faster retrogradation of starch during film formation could lead to lower opacity of the films. Shah et al. [36] reported that a less opaque film implies higher transparency. This study explored that the addition of EO in the edible film composition had maintained the lack of opaqueness. The edible films’ visual appearance is a vital tool in the food packaging industry to influence consumers’ perception of a food product. Overall, the present study exhibited a smooth appearance of the film surface in all the samples (Figure 2A–E). The films with and without the addition of EOs showed no evidence of cracks, pores, or fissures. There were not many differences in appearance between the front-facing and backside of the films; however, the front-facing side of the film was slightly smoother compared to the casting side. Normally, chitosan-based edible films exhibit a slightly light brown color [37]. This study found that regardless of the EO incorporation in the chitosan/NGRS-based edible film, the overall color appearance was not changed among them. The transparency of the films was minimal, and it is due to the higher opacity of the films. This is in accordance with the study of Mohamad et al. [38]. Bonilla et al. [39] reported that the incorporation of EOs improved the smoother surface of the film, and the smoother surface could make the films glossier, and their study also suggested that composite films are less glossy than pure chitosan films.

### 3.2. Thickness and Textural Properties

**Figure 3:**
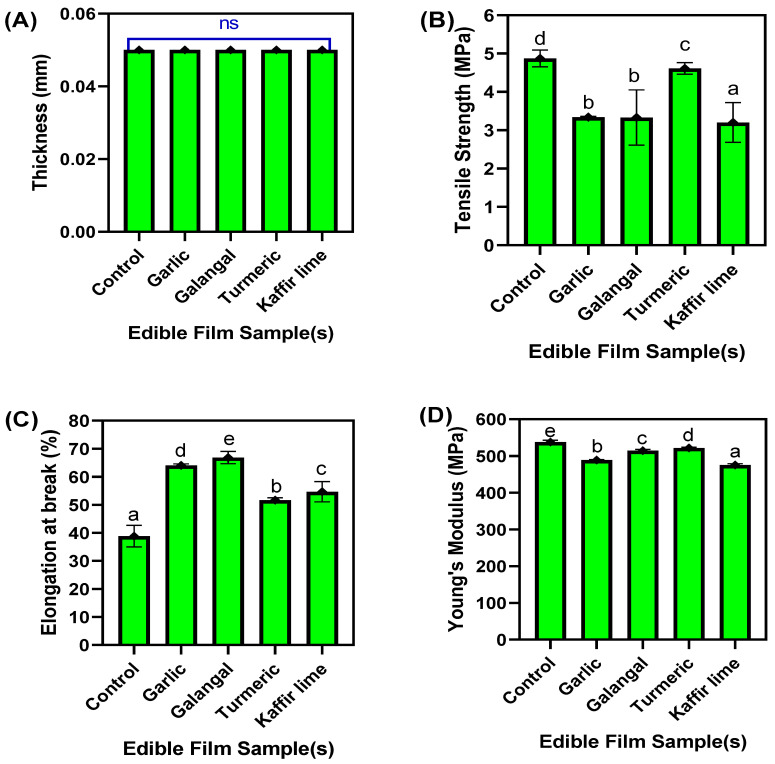
Thickness (**A**), tensile strength (**B**), elongation at break (**C**), and Young’s modulus (**D**) of chitosan–NGRS based edible film incorporated with and without EOs. Note: The different letters shown in the figure represents significant differences between means; ns represents non-significance.

The thicknesses of the edible films made of chitosan and NGRS and incorporated with various EOs were tested, and the results are given in Figure 3A. Thickness is one of the significant characteristics of edible films and coatings that greatly affect the biological nature and shelf life of food products due to its influence on other factors composed of water, optical, and textural characteristics [40,41]. Santacruz et al. [42] reported that the increased concentration of starch content with the high swelling power in the edible film composite might increase the film’s thickness. Furthermore, a higher level of hydrophilic plasticizers such as glycerol could likely retain more moisture in the film matrix and thus increase the film thickness [41]. The tested samples in this study showed that the film thickness was not significantly different. It demonstrated that chitosan/NGRS-based composite film could form a thin film within the range of 0.0495 to 0.0505 mm. Edible films with 0.050 mm of thickness are commonly referred to as thin and cohesive film layers [43]. Skurtys et al. [44] suggested that the ideal thickness of the edible films should be less than ≤0.25 mm. The present study is within the recommended thickness range of the biopolymer-based edible films. Textural properties such as tensile strength, elongation at break, and Young’s modulus of the tested edible films are shown in Figure 3B–D. Despite the film differences, the tested edible films’ tensile strength and elongation at break were significantly affected. Overall, a higher level of tensile strength was noticed in the chitosan/starch-based edible film with no EO, followed by the EO-added films. According to Liu and Han [45], starches with a high amylose content produce solid and flexible films. The present study found that the addition of EOs significantly affected the film’s tensile strength compared with the control. Among the EO-added films, the garlic, galangal, and kaffir lime EO-added films showed no significant differences as compared to turmeric EO, which showed the highest tensile strength. On the other hand, the elongation at the break of the tested films exhibited a contrary effect against the tensile strength. The least elongation at break was noticed in control films, indicating lesser flexibility, followed by the EO-added samples. Overall, the EO-added film samples exhibited slightly more elongation strength. Among the samples, the galangal and garlic EO-added films showed higher elongation at break. Yekta et al. [46] reported that the addition of EOs in the film could induce intense chemical reactions between the functional groups of the EOs and the film polymer, thus inducing the efficient transfer of stress throughout the polymer layers and creating stronger film elasticity. Sutput et al. [47] also found a similar trend of increased tensile strength having significantly decreased the elongation at break values in the starch-based edible film upon the addition of EOs. Young’s modulus (YM) or elastic modulus, a measure of stiffness, indicates optimum stiffness by a higher value of YM [48]. The YM values of the tested edible film indicate that the overall stiffness of the films is at moderate levels. Compared with the control film, the YM value of the EO-added films was significantly low. Among the EO-added films, the kaffir lime and garlic EO-added film samples exhibited slightly lesser stiffness than the other EO-added films. Buso-Rios et al. [49] also observed lower levels of YM in the starch-based biodegradable film that contained EOs.

### 3.3. Moisture Content and Water Vapor Permeability

**Figure 4:**
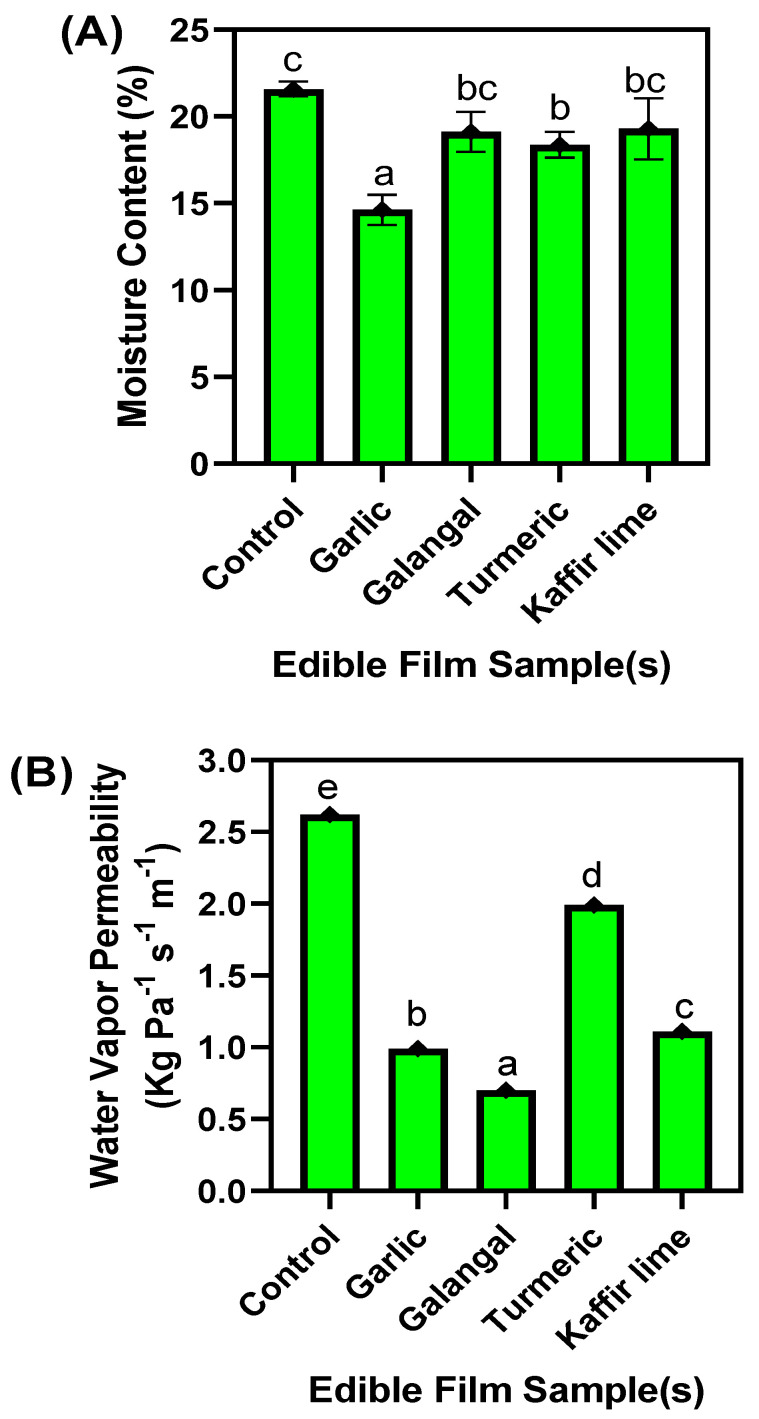
Moisture content (**A**) and water vapor permeability (**B**) of chitosan–NGRS based edible film incorporated with and without EOs. Note: The different letters shown in the figure represents significant difference.

The moisture content (MC) and water vapor permeability (WVP) of the edible films made of chitosan and NGRS and incorporated with various EO were tested, and the results are given in Figure 4A,B. MC is one of the key characteristics of edible films, as it heavily influences the film’s mechanical properties [48]. The MCs in the tested films significantly differed; overall, the highest level of MC is recorded in the chitosan/starch-based edible films. Glycerol is the key base material, adversely influencing the film’s moisture content [34]. An increased glycerol concentration significantly increased the MC and WVP level in the films [50] because the glycerol contains the free hydroxyl group. It could bind with water via hydrogen bonding and thus increase the MC in the films [51]. The EO-added film samples were slightly lowered than the control and, among them, galangal and kaffir lime EO-added film samples contained slightly higher moisture, followed by the garlic and turmeric EO-added films. In comparison with all the samples, the turmeric EO-added edible films retained the lowest moisture level. Abdollahi et al. [52] reported that addition of EOs increased the hydrophobicity of the film and thus prevented external moisture observation and performed better in preventing WVP. This study found that the MC and WVP of the films were irrelevant to each other. Though the control samples were high in WVP, similar to MC, when compared among the EO-added film samples, the WVP and MC of EO-added films samples significantly varied. Turmeric EO-added film samples were high in WVP followed by kaffir lime, garlic, and galangal EO-added film samples. Phan et al. [53] found that film compositions with high starch content, particularly low amylose content, could adversely increase the film’s WVP. Santacruz et al. [42] found that the use of glycerol as plasticizer increased the WVP level to a high compared to other types of plasticizers. Madhavi et al. [54] reported that addition of EOs to the film forming solution significantly controlled WVP in the film. Anis et al. [55] reported that the film thickness could also adversely affect the WVP, and the addition of EOs in the polymer matrices could somewhat increase the thickness and thus reduced the WVP.

### 3.4. FTIR Spectra

**Figure 5:**
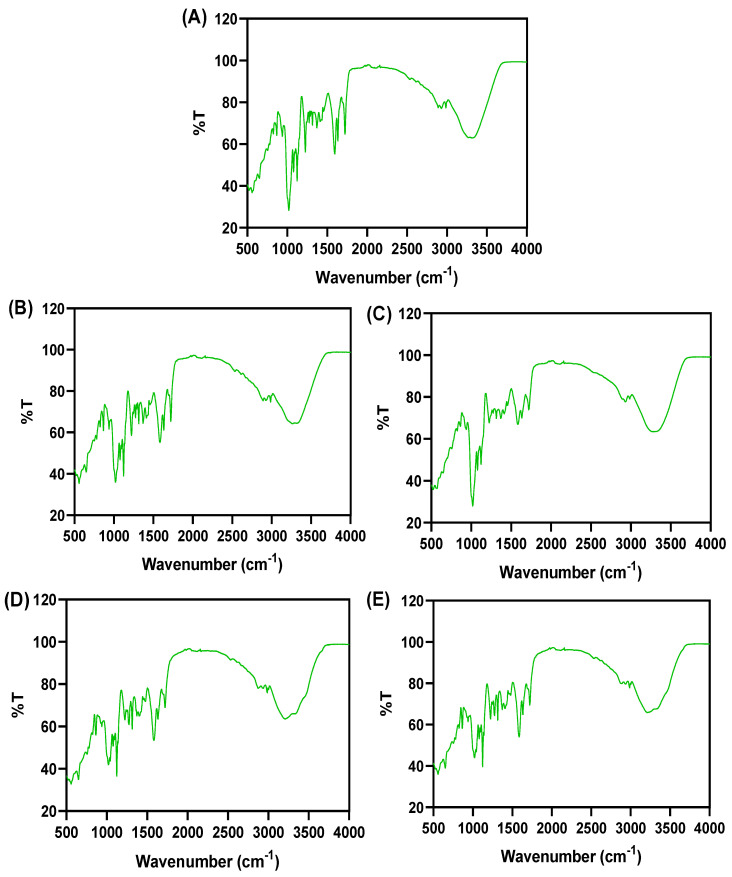
FTIR spectra (**A**–**E**) of chitosan–NGRS based edible film incorporated with and without EOs. (**A**) represents control film; (**B**) represents garlic oil added film; (**C**) represents galangal oil added film; (**D**) represents turmeric oil added film; (**E**) represents kaffir lime oil added film.

FTIR spectra of the edible films made of chitosan and NGRS and incorporated with various EOs are shown in Figure 5. Normally, the interaction between the polysaccharide food-based components that connect with the additives or other related chemical components by bonding, specifically hydrogen bonding, is tested by the FTIR spectra, which are a rapid, nondestructive, and powerful spectroscopy tool [56]. Overall, despite the EO types, the FTIR spectra observed a broadband between 1000 and 1700 cm^−1^ in the chitosan and starch-based film, and it could be due to the formation of hydrogen bonding between starch and non-starch polysaccharide and glycerol during film formation [57]. Overall, there were not many differences in the profile shape of the transmittance in the FTIR spectra (558, 647, 867, 1019, 1122, 1222, 1313, 1582, 1632, 1720, 2984 cm^−1^) observed for the tested chitosan and NGRS based edible film samples compared with the other film samples that had EOs. However, the differences in the peak intensity for films with different EOs varied significantly. This is in accordance with the study of Nobrega et al. [58], where they observed similar FTIR spectra of the base film, despite the differences in the samples. All the tested films had the wider broadbands between 3200–3500 cm^−1^. This is in accordance with the study of Zhou et al. [59]. Abdul Ghani et al. [57] reported that chitosan-based edible film displayed a broadband at 3410 cm^−1^, corresponding to the extended hydroxyl (O-H) stretching vibration by hydrogen bond. Davoodi et al. [60] observed FTIR broadbands ranging from 3240 to 3410 cm^−1^ in a polysaccharide based biodegradable edible film, and they also suggested that peak at this level represented the stretching O-H between the film forming material, particularly hydrogen bonding within the polymers, or within plasticizer and/or between polymer and plasticizer, and/or between plasticizers and with the bound water in the film. Zhang et al. [61] reported that broadband observed at a level of more than 3000 cm^−1^ represents the hydrophilicity of the polymer. The peak values found between 1600 and 1700 represent the C=C stretching whereas the peaks found at a level of 2984 cm^−1^ could represent the C-H stretching [57,61,62]. Choudhury et al. [63] reported that the weaker absorbance band found within the range of 2900 cm^−1^ represented the presence of C-H vibration of the methyl group. Ning et al. [64] suggest that the band at 1018 cm^–1^ is a characteristic peak of the C-O bond in the glucose ring, and that 1080 and 1101 cm^−1^ are characteristic peaks of the C-O bond in the C-O-H group of starch. There are characteristic stretching bands in C-O between 1160 and 930 cm^−1^, which appear in polymer and starch. Galangal EO-incorporated edible film had a peak absorbance broadband at 1021 cm^−1^ and 1125 cm^−1^, indicating the absorbance of C-OC and CO-. This is in accordance with the study of Zhou et al. [59]. The control and galangal EO-added edible films had almost similar pattern of peaks in the FTIR spectra, indicating that there was no interaction between the active components from galangal EO and chitosan and native glutinous starch-based film, whereas garlic, kaffir lime and turmeric EO-added films exhibited differences in the peaks. Furthermore, a comparison between the kaffir lime and turmeric EO-added films also exhibited no differences between them in the peaks of broadbands, and it could confirm that the active components from both EOs had a similar pattern of interactions with the tested edible film. Pranoto et al. [16] tested different concentrations of garlic EO on the chitosan based edible film, and their findings showed no interconnection between the film polymer and EO. Overall, the FTIR spectra observation of the edible film showed that application of garlic EO and Kaffir lime EO or turmeric EO had little effect on the functional group change of the chitosan/NGRS based edible film, and it did not show any significant impact on the film in improving the physical properties in comparison with the control film.

### 3.5. Microstructural Observations

**Figure 6:**
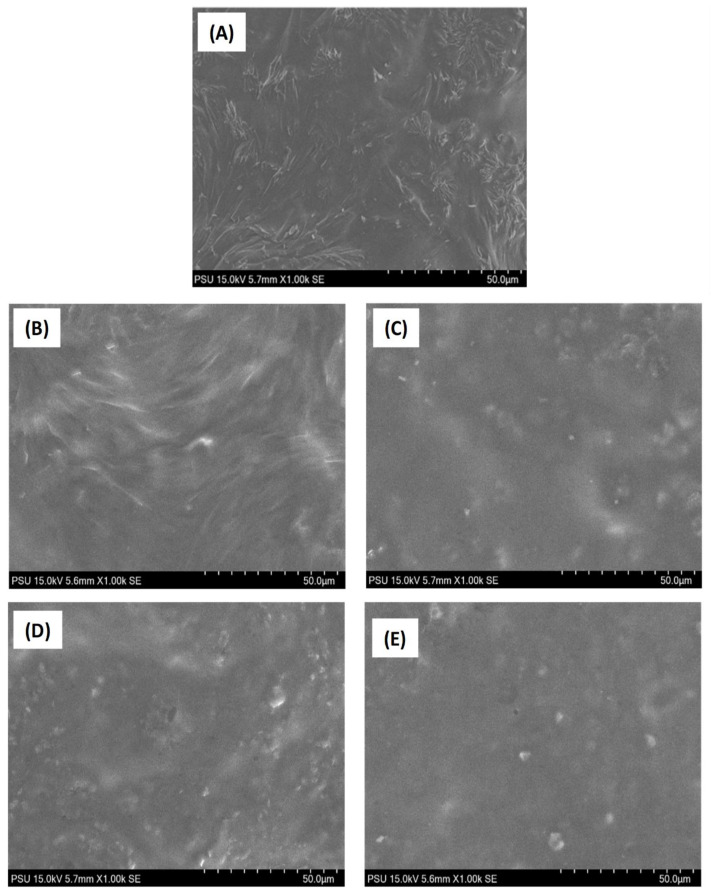
Microstructural observation of the surface section (**A**–**E**) of chitosan–NGRS based edible film incorporated with and without EOs. (**A**) represents control film; (**B**) represents garlic oil added film; (**C**) represents galangal oil added film; (**D**) represents turmeric oil added film; (**E**) represents kaffir lime oil added film.

**Figure 7:**
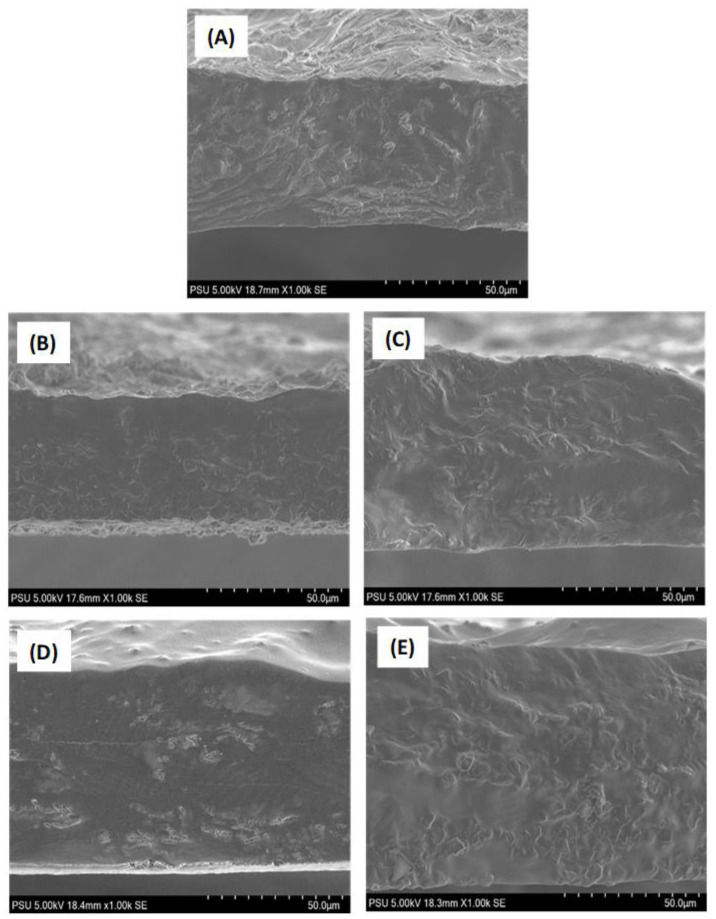
Microstructural observation of the cross-sections (**A**–**E**) of chitosan–NGRS based edible film incorporated with and without EOs. (**A**) represents control film; (**B**) represents garlic oil added film; (**C**) represents galangal oil added film; (**D**) represents turmeric oil added film; (**E**) represents kaffir lime oil added film.

The microstructural observation of the surface and cross-section of the edible films made of chitosan and NGRS and incorporated with various EOs is shown in Figure 6 and Figure 7. Microstructure analysis was performed qualitatively to demonstrate the role of non-starch- and starch-based polysaccharide edible film interconnected with the different EOs. Generally, the microstructural changes in the edible films depend on the procedures applied to prepare the film and the ingredient and drying process [65]. The surface and cross-section of tested edible films exhibited various microstructural patterns and the additions of EOs significantly influenced it. Overall, the surface sectional observation of all the edible films presented a holeless and fissureless structure. Films without EOs demonstrated an irregular rough microstructural pattern. This could be due to the evaporation of moisture during film preparation. Amalraj et al. [66] also observed a similar finding that chitosan-based film evaporated of moisture during preparation resulted in an irregular rough surface. On the other hand, the EO-incorporated films in this study showed homogenous smooth patterns in both surface and cross-sections. This was applicable to all the varieties of EOs used in this study. Among the different EOs, the galangal EO-based films were much smoother compared to kaffir lime EO, followed by the EOs from garlic and turmeric. Normally, the smoother observation of edible film microstructure indicates proper incorporation of EOs in the film matrix. The present study found that adding galangal EO could improve the film microstructural appearance. It might be because of the smaller galangal EO droplet size which allowed an increase in the interactions between the polymer matrix and the oil, and resulted in a less cohesive chitosan and NGRS matrix. This finding is an accordance with the study of Aljobair [67]. Sapbhon et al. [68] tested the non-starch-polysaccharide-based edible incorporated with kaffir lime EO. Their results showed that addition of EO formed microdroplets in the film emulsion and thus created a smooth surface and cross-sectional microstructure. Furthermore, their study also proved that kaffir lime EO was bound in the film and able to release the fragrance slowly for six months. Zhao et al. [69] tested the microstructural observation of garlic EO-based emulsion, and their report showed that garlic EO displayed a smaller droplet and uniform dispersion in the emulsion. It is indicated that addition of garlic EO in the edible film-based emulsion could enhanced the even dispersibility; however, it could adversely affect the film morphology if the affinity of the EO and film forming materials interacted well. Yusof et al. [70] studied the microstructural observation of chitosan-starch-based edible film with turmeric EO, and their findings reported that adding turmeric EO in the film composition created a homogenous and highly miscible film surface with slightly rough cross-sections. Additionally, despite the EO types, the surface sectional observation showed that the edible film incorporated with EO had numerous small patches all over the film surfaces. This could be due to moisture disturbance in the film, as it tries to escape during the drying process, and the EOs in the films could have influenced this action. Espitia et al. [71] found similar findings that non-starch-based edible films incorporated with EO created a heterogenous, bumpy, and crater-like structure in the film when the microstructural observations of the surface sections were examined. However, galangal and Kaffir lime EO-added edible films had the lowest surface disturbance compared to others. Zhao et al. [69] found that chitosan-based edible films incorporated with EO reduced bubble formation and porosity, and improved the structural strength of the films. Yeddes et al. [72] reported that irregularities of the edible film’s microstructure are influenced by the thickness of the film as well as the concentration of EO. The present study used similar concentration of EOs for all the film preparations but still observed the irregular pattern in the film’s surface section. This could be due to the different compositions of EOs that slightly influenced the film’s appearance. This is in accordance with the study by Sedlarikova et al. [73]. Furthermore, weak interaction between the chitosan and native starch-based edible films and EOs with high active components could induce a higher coalescence between EO globules, thus creating irregular film patterns [16,55,74].

### 3.6. Antimicrobial Properties

Antimicrobial activities against food pathogens including *E. coli*, *S.* Typhimurium, *L. monocytogens*, *S. aureus*, *P. fluorescens*, and *L. plantarum* using edible films made of chitosan and NGRS and incorporated with various EOs were tested and the results are given in Table 1. The antimicrobial activities of the edible films with and without EOs showed significant effects against food-born pathogenic bacteria. Furthermore, the intensity of the activities against the pathogens varied vastly based on the EO. The overall results showed that the inhibitory effect of the films was noticed in all the samples at 24 h, though some of the EOs showed an inhibitory effect from 2 h, of the incubation period. Overall, the control films, which had chitosan and NGRS, showed antimicrobial activities against all tested pathogens. Overall, the noticeable level of activity from control films was established after the microbial incubation period was kept for 12 h. Then the severity of the inhibitory effect increased every 12 h. Control films could inhibit *E. coli* completely within 48 h, followed by *L. monocytogenes* and *S.* Typhimurium, which showed some growth at 48 h. Control samples did not effectively suppress the growth of other microorganisms tested in this study. Normally, the broad antimicrobial spectrum of chitosan makes it highly effective against all types of bacteria, including gram-positive, gram-negative, and fungi. Goy et al. [75] suggested that chitosan could control microbial growth with three possible mechanisms: ionic surface interaction induced cell leakage, inhibition of mRNA and protein synthesis, and lastly formation of an external barrier to chelate nutritional supply for microbial growth. Luan et al. [76] found that different molecular weights of chitosan exhibited a significant role in controlling microbial growth. Their study suggested that higher kilodaltons of chitosan showed excellent performance compared to lower ones. Ardean et al. [77] reported that despite the chitosan concentration inhibiting bacterial growth, acetylation should be considered as it equally plays a major role in suppressing microbial growth. Guarnieri et al. [78] found that the application of chitosan plays a significant role in controlling fungi growth over bacteria. Among the EO-added films, the garlic and galangal EO showed excellent inhibitory effects against most of the tested pathogens in this study. Garlic EO-added films took 12 h of incubation time to kill all the *E. coli*, followed by films with galangal EO. On the other hand, turmeric and kaffir lime EO-added films were able to reduce the number of *E. coli* but could not eliminate *E. coli* within the tested period. Garlic EO incorporated in the edible film made of alginate showed a greater inhibitory effect against gram-positive bacteria as compared with the gram-negative bacteria; however, when garlic EO incorporated with the chitosan-based film showed inhibitory activity it was mainly due to the additional contribution by the innate characteristics of the antimicrobial activity of the chitosan [16,79]. *S. Typhimurium* count was significantly reduced within 4 h of incubation and complete inhibition by garlic EO-added films was evidenced within 8 h incubations. On the other hand, the galangal, turmeric, and kaffir lime EO-added films were only able to reduce the growth of *S.* Typhimurium but could not eliminate it. Similar results were also observed in a study by Perdana et al. [80], which found that kaffir lime EO-added starch/chitosan film poorly controlled the *S.* Typhimurium. In the case of *L. monocytogenes*, among the EO-added edible films, the kaffir lime EO very effectively controlled the growth of *L. monocytogenes*; a complete inhibitory effect of kaffir lime EO was noticed from 8 h after incubation, whereas the other samples started to exhibit complete control of this pathogen at the 24–48 h period of incubations. However, turmeric and galangal EO-added films performed slightly better than garlic EO-based film and the control film. Kaffir lime EO contains rich sources of sabinene, limonene, citronellal, and β-pinene, which are excellent sources for inhibiting *L. monocytogenes* [81]. On the other hand, compared with garlic and galangal EO-added films, the performance of kaffir lime and turmeric based EO films was poor in terms of exhibiting a complete inhibitory action against *S. aureus*. Galangal EO-based film exhibited a significant antimicrobial activity against *S. aureus* at 8 h of incubation, and similar activity was noticed at 12 h in the garlic EO-based films. Mayachiew and Devahastin [82] reported that galangal is an excellent source of terpenes, a crucial organic compound needed for inhibiting *S. aureus*. Seydim et al. [83] found that garlic essential oil added to the protein-based edible significantly controlled the growth of *S. aureus* in sliced Kasar cheese. Similarly, the growth of *P. fluorescens* was also significantly inhibitory to the garlic and galangal EO-based edible films compared to other samples. Garlic and galangal EO could completely inhibit the *P. fluorescens* growth at a 24 h period of incubation. In contrast, the other samples could not completely inhibit *P. fluorescens* throughout storage. On the other hand, the *L. plantarum* growth count could not be controlled by any of the edible film samples regardless of the EOs used; however, the inhibitory effect of the films slowly rose as the incubation period extends. Overall, turmeric and kaffir lime EO-based films could control the complete growth of *L. plantarum* at 48 h of incubation. In comparison to kaffir lime EO-based films, turmeric EO films performed slightly better. The other tested films could not completely inhibit the *L. plantarum* in the given incubation period. Overall, the essential-oil-incorporated chitosan/rice-native-starch-based edible film showed good inhibitory activity against the tested pathogens; however, their efficacy in controlling the pathogens varied significantly among the EOs and this difference could be caused by the biopolymeric matrix. The chitosan-and-NGRS-based biopolymer edible film matrix could have been impaired in releasing the active compounds from essential oils in the given time period, thus adversely affecting the overall performance of the EOs. This is in an accordance with the study of Gomez-Estaca et al. [84].

## 4. Conclusions

The current study shows that incorporating essential oils into biopolymer-based edible films made of chitosan and native glutinous rice starch has great potential as a food packaging material and can act as an antimicrobial agent to inhibit the growth of both gram-positive and gram-negative foodborne pathogens. The addition of essential oils improves the color, appearance, hydrophobic properties, and mechanical properties such as tensile strength and elongation at break of the film. Among the different essential oils, turmeric essential-oil-incorporated edible films exhibited better physical and mechanical properties. Analysis of the film’s Fourier-transform infrared spectrum and microstructure observation showed that the essential oils interacted with the biopolymers’ functional groups, resulting in a smooth and sturdy film and, among all the samples, the galangal and kaffir lime essential-oil-added films exhibited better microstructural appearance. This study found that addition of garlic, galangal, and turmeric EOs into the biopolymer composite edible film could diminish the pathogenic bacteria completely. Overall, this study suggests that chitosan and native glutinous rice starch are effective for creating edible films and, when combined with essential oils, can provide a long-term solution for storing dry and fresh foods without bacterial growth. Among the different essential oil used, this study recommends garlic and galangal essential oils as active ingredients to apply in polysaccharide based composite film for overall performance as protective agents for preserved foods.

## Figures and Tables

**Table 1:** Antimicrobial activity of chitosan–NGRS based edible film incorporated with and without EOs.

Treatment	Amount of *E. coli* Count (CFU/mL)						
	0 h	2 h	4 h	8 h	12 h	24 h	48 h
Control	TNTC	TNTC	TNTC	TNTC	TNTC	173.33 ± 23.50	0.00 ± 0.0
Garlic	TNTC	TNTC	TNTC	TNTC	0.00 ± 0.0	0.00 ± 0.0	0.00±0.0
Galangal	TNTC	TNTC	TNTC	TNTC	TNTC	25.03 ± 1.0	0.00 ± 0.0
Turmeric	TNTC	TNTC	TNTC	TNTC	TNTC	65.00 ± 0.0	45.00 ± 0.0
Kaffir lime	TNTC	TNTC	TNTC	TNTC	TNTC	30.00 ± 0.0	21.67 ± 19.3
**Treatment**	**Amount of *S.* Typhimurium count (CFU/mL)**						
	0 h	2 h	4 h	8 h	12 h	24 h	48 h
Control	TNTC	TNTC	TNTC	TNTC	TNTC	89.33 ± 48.7	28.50 ± 19.0
Garlic	TNTC	TNTC	74.00 ± 0.0	0.00 ± 0.0	0.00 ± 0.0	0.00 ± 0.00	0.00 ± 0.0
Galangal	TNTC	TNTC	TNTC	TNTC	TNTC	230.00 ± 0.00	61.00 ± 0.0
Turmeric	TNTC	TNTC	TNTC	TNTC	TNTC	65.00 ± 0.00	25.67 ± 9.3
Kaffir lime	TNTC	TNTC	TNTC	TNTC	TNTC	60.00 ± 0.00	35.60 ± 19.0
**Treatment**	**Amount of *L. monocytogenes* count (CFU/mL)**						
	0 h	2 h	4 h	8 h	12 h	24 h	48 h
Control	TNTC	TNTC	TNTC	TNTC	TNTC	246.00 ± 25.9	21.67 ± 9.4
Garlic	TNTC	TNTC	TNTC	TNTC	TNTC	83.00 ± 11.0	0.00 ± 0.0
Galangal	TNTC	202.00 ± 0.0	116.00 ± 1.3	68.00 ± 7.0	10.00 ± 2.8	0.00 ± 0.0	0.00 ± 0.0
Turmeric	TNTC	22.33 ± 6.0	17.00 ± 6.9	16.00 ± 8.5	13.67 ± 6.03	0.00 ± 0.0	0.00 ± 0.0
Kaffir lime	TNTC	TNTC	TNTC	0.00 ± 0.0	0.00 ± 0.0	0.00 ± 0.0	0.00 ± 0.0
**Treatment**	**Amount of *S. aureus* count (CFU/mL)**						
	0 h	2 h	4 h	8 h	12 h	24 h	48 h
Control	TNTC	TNTC	TNTC	TNTC	TNTC	183.00 ± 48.6	170.00 ± 83.4
Garlic	TNTC	132.00 ± 0.0	100.00 ± 4.2	57.00 ± 0.0	1.33 ± 1.5	0.00 ± 0.0	0.00 ± 0.0
Galangal	TNTC	TNTC	TNTC	7.00 ± 5.6	1.00 ± 0.0	0.00 ± 0.0	0.00 ± 0.0
Turmeric	TNTC	TNTC	TNTC	TNTC	TNTC	40.00 ± 0.0	2.33 ± 0.2
Kaffir lime	TNTC	TNTC	TNTC	TNTC	TNTC	25.00 ± 0.0	7.50 ± 6.3
**Treatment**	**Amount of *P. fluorescens* count (CFU/mL)**						
	0 h	2 h	4 h	8 h	12 h	24 h	48 h
Control	TNTC	TNTC	TNTC	TNTC	TNTC	TNTC	176.00 ± 31.1
Garlic	TNTC	TNTC	44.00 ± 0.0	17.33 ± 4.1	6.00 ± 5.2	0.00 ± 0.0	0.00 ± 0.0
Galangal	TNTC	202.00 ± 0.0	179.00 ± 0.0	125.01 ± 3.1	80.00 ± 0.0	0.00 ± 0.0	0.00 ± 0.0
Turmeric	TNTC	TNTC	TNTC	TNTC	TNTC	166.21 ± 5.4	85.10 ± 3.2
Kaffir lime	TNTC	TNTC	TNTC	TNTC	TNTC	TNTC	69.23 ± 11.0
**Treatment**	**Amount of *L. plantarum* count (CFU/mL)**						
	0 h	2 h	4 h	8 h	12 h	24 h	48 h
Control	TNTC	TNTC	TNTC	TNTC	169.00 ± 17.69	156.67 ± 38.44	143.00 ± 13.7
Garlic	TNTC	TNTC	TNTC	TNTC	106.00 ± 27.92	85.50 ± 20.51	14.50 ± 3.5
Galangal	TNTC	TNTC	TNTC	TNTC	81.66 ± 7.11	39.00 ± 0.0	21.00 ± 8.4
Turmeric	TNTC	TNTC	TNTC	91.00 ± 0.0	43.00 ± 11.54	2.00 ± 0.00	0.00 ± 0.0
Kaffir lime	TNTC	TNTC	TNTC	191.00 ± 1.3	85.50 ± 5.55	23.21 ± 3.14	0.00 ± 0.0

Note: TNTC indicates too numerous to count.

## Data Availability

Not applicable.
